# Memory rehabilitation during the COVID-19 pandemic

**DOI:** 10.1186/s12911-023-02294-1

**Published:** 2023-09-27

**Authors:** José Luis Varela-Aldás, Jorge Buele, Doris Pérez, Guillermo Palacios-Navarro

**Affiliations:** 1 Centro de Investigaciones de Ciencias Humanas y de la Educación (CICHE), Universidad Indoamérica, Ambato, Ecuador; 2 Department of Electronic Engineering and Communications, University of Zaragoza, Teruel, Spain; 3 Carrera de Psicología, Facultad de Ciencias de la Salud y Bienestar Humano, Universidad Indoamérica, Ambato, Ecuador

**Keywords:** Cognition, Cognitive training, COVID-19, Episodic memory, Longitudinal studies, Mobile applications

## Abstract

**Background:**

Loss of cognitive and executive functions is a problem that affects people of all ages. That is why it is important to perform exercises for memory training and prevent early cognitive deterioration. The aim of this work was to compare the cognitive performance of the participants after an intervention by using two mnemonic techniques to exercise memory functions (paired-associate learning and method of loci).

**Methods:**

A longitudinal study was conducted with 21 healthy participants aged 18 to 55 years over a 2-month period. To assess the impact of this proposal, the NEUROPSI brief battery cognitive assessment test was applied before and after the intervention. In each session, a previous cognitive training was carried out using the paired-associate learning technique, to later perform a task based on the loci method, all from a smart device-based application. The accuracy response and reaction times were automatically collected in the app.

**Results:**

After the intervention, a statistically significant improvement was obtained in the neuropsychological assessment (NEUROPSI neuropsychological battery) reflected by the Wilcoxon paired signed-rank test (*P* < .05).

**Conclusion:**

The task based on the method of loci also reflected the well-known age-related effects common to memory assessment tasks. Episodic memory training using the method of loci can be successfully implemented using a smart device app. A stage-based methodological design allows to acquire mnemic skills gradually, obtaining a significant cognitive improvement in a short period of time.

## Background

Memory is a cognitive system with a high degree of complexity that allows the human being to encode, store, retain and recall information [1, 2]. This is one of the cognitive functions that man has more developed compared to animals. It is a basic element of the learning process, which is based on two main storage categories: short-term and long-term [3]. However, memory should not be viewed as a single entity. Cognitive psychology and neuropsychology theories have described more than 30 different types of memory, distributed in different classifications [4]. These allow knowing the meaning of words, remembering events, historical facts, learning new skills, associating elements, etc. [2]. These associations can be simple, such as relating an object to a word or a face to its name, as well as more complex ones, because one memory is often combined with others. This process involves several brain regions such as the frontotemporal regions, the hippocampus, and the temporal poles [5]. In particular, episodic memory allows the encoding and retrieval of information regarding a place, emotion, specific time and life episodes with clear recall [6]. In the work of Sandberg et al. it is mentioned that the performance of this memory is sensitive to age, although other factors could cause it to vary in each individual [7].

There are different alterations that can affect memory, which decreases the person’s quality of life [8, 9, 10]. When talking about episodic memory, drawbacks in recalling memories can be identified in both young and old people [11, 12]. Problems such as concentration can manifest, with forgetfulness about conversations that are taking place when there is a slight distraction. This can limit the ability to learn new skills, affect the retention of intended information [13] and impede the performance of activities of daily living (ADL) [14]. Among its main symptoms can be mentioned the difficulty in expressing oneself adequately orally or in writing, repeating words or phrases repeatedly [15]. The causes of memory problems are varied and can include physiological and emotional factors. For example, the appearance of Mild Cognitive Impairment (MCI), a condition that can be part of normal or pathological aging and is often associated with memory difficulties and other cognitive processes [16]. Other causes include emotional or psychological traumatic events, depression or anxiety, or the consumption of certain medications. It can also be generated after having suffered a Cerebrovascular Accident (CVA), epileptic attacks or be the result of constant aneurysms and encephalitis [17].

Since the first official outbreak of COVID-19 in December 2019, the scientific community has devoted considerable effort to understanding the neuropsychological and respiratory syndrome-associated consequences of the disease [18]. According to a meta-analysis conducted in 2021, there was an increase of 26.9% in cases of major depressive disorders and 27.8% in cases of anxiety disorders worldwide [19]. This pattern is consistent with what was presented in a meta-analysis [20], which demonstrated a considerable increase in mental health symptoms during COVID-19. Research published in The Lancet mentions that most people who have suffered negative life events, such as bereavement or exposure to disasters, show resilience (minimal impact on symptoms of anxiety or depression, or both) or recovery (an initial short-term increase in symptoms of anxiety, depression, or both, followed by recovery) [21]. These symptoms diminished over time, but demonstrated that humans are vulnerable to psychological disorders that could exacerbate problems related to cognitive decline and dementia.

In order to identify the appearance of problems in human cognition, several tools have been developed for their evaluation [22]. Selection of the appropriate tool depends on the specific needs of the patient, the nature of the injury, and the extent of cognitive impairment. Standard measures include comprehensive neuropsychological batteries, tests that assess the impact on daily life, memory questionnaires, and specific subtests [23]. It must be recognized that the loss of memory capacity not only involves memory problems, but also the use of other executive and cognitive functions. To effectively retain information for a short period of time or to retrieve it from the past, proficient encoding, manipulation, and retrieval are required. That is why, after identifying these deficits, memory training programs should be implemented to strengthen it, as well as the complementary functions involved.

There are cognitive training processes that have been designed and implemented since classical times. The Method of Loci (MoL) or memory palace method takes advantage of the brain’s ability to spatially organize both concepts and thoughts [24]. This mnemonic strategy is based on a spatial metaphor, in which the user is asked to imagine a known palace or building and navigate their minds through it [25, 26]. By associating this imagined structure with specific things to remember, this method has demonstrated that memory is influenced by environment and context. Memory retention is enhanced when one imagines being in the place where the learning occurred. As this strategy requires a high degree of concentration, it promotes extensive cognitive work and causes changes in the pattern of brain activation, as reported by Kondo et al. [11].

Active navigation within these mental palaces depends on their construction and subsequent recall [24, 27]. As a result, along with episodic memory, this technique requires spatial memory operation and verbal recall performance, offering comprehensive cognitive training [28]. The MoL has now been modernized and computerized as can be seen in Caplan et al. [25] or Sandberg et al. [7]. In Sandberg et al. [7] a MoL-based smartphone app was used for episodic memory training in young and old adults. Similarly, Gross et al. [29] carried out a study using 5 years of longitudinal follow-up to assess that after training to encourage the use of the MOL technique, the group of participants who chose to use this strategy, associated with immediate memory improvement. Such improvement was sustained through the follow-up period. Huttner et al. [30] implemented the MoL in a virtual memory palace, whose results showed that its application in virtual environments could reduce the mental load for younger learners as well as motivate them. Lastly, a new variant of implementation of the MoL has been taken to the knowledge retrieval form scholarly articles instead of remembering a list of items with quite good results [31]. Although previous studies support a good adaptation of the MoL in virtual environments, there are studies such as the one conducted by Vindenes et al. [32] who stated that the MoL could not benefit from this adaptation and technological help. In fact, those learners with low spatial ability might experience more cognitive overload than others.

The situation presented exposes the need for integrated e-health solutions in the field of cognitive rehabilitation. Due to the global impact of COVID-19, access to cognitive rehabilitation therapies that can be delivered anywhere is essential now more than ever. Smartphone apps, in particular, offer a potentially effective and accessible solution for almost everyone. They allow users to perform memory exercises and track their progress from the safety of their homes, thus relieving some of the stress associated with the pandemic [33, 34].

### Our contribution

Our study adds an important novelty that lies in the way of implementing a memory task and administering the intervention, with the inclusion of a relaxation period in the cognitive training pass, as well as the inclusion of a task prior to the main memory task (MoL). In the first case, the relaxation sessions improve the participant’s concentration and attention, since it has been shown that breathing exercises favor the use of cognitive functions. Second, the previous implementation of a classical task widely used for memory rehabilitation with a lower level of complexity, prepares the participants for the subsequent task. Different authors have pointed out the need to perform approximation tasks when you want to use a task that demands a lot of cognitive activity [31].

On the other hand, in the aforementioned MoL approaches, visuospatial spatial environments have been used to scaffold the memorization of non-spatial information, especially through implementations in Virtual Reality (VR) systems. Unlike these studies, in our case we have implemented the MoL in a simple application for low-cost smart devices to be used for memory training and cognitive rehabilitation purposes. We hypothesized that after applying a novel methodological approach based on three phases (relaxation, pre-training and training with MoL) for a period of 2 months, the participants will show significant improvements in cognitive performance.

## Methods

### Study design

The study is quantitative quasi-experimental with a longitudinal pre-test and post-test design; whose objective was to train memory through 2 traditional mnemonic techniques implemented in an application for a smart device. The study was performed in accordance with the ethical standards as laid down in the 1964 Declaration of Helsinki and its later amendments. The protocol used in this study was approved by the Research Ethics Committee of the Universidad Indoamérica on 4th January 2022 (protocol IIDI-002) with the understanding and written consent of all the participants before the study.

The research began with the recruitment of volunteers, and we sought patients without a diagnosis of any neurodegenerative disease, or a history of memory problems. The group of participants underwent an initial neuropsychological assessment to determine their cognitive status. After this assessment, the cognitive training intervention was started for a period of 2 months. Data on Accuracy Response (AR) and task completion time or Reaction Time (RT) were collected automatically using a smart device-based application. The same cognitive assessment battery was applied after the intervention.

### Sample

The sample was non-probabilistic for convenience, because the study requires a commitment from the participants to attend the sessions on established dates. The participants were recruited from a group of volunteers in the city of Ambato, Ecuador and did not receive any financial benefits for their participation in the study. The inclusion criteria for the participants were voluntary acceptance through informed consent, without gender discrimination, age between 18 and 60 years, with at least 10 years of formal education, and obtaining scores of 4 for the recall of verbal information and 8 0.5 for visuospatial evocation of figure [35]. Exclusion criteria were: having neurological diseases or cognitive problems, intellectual disability or being under the influence of psychotropic substances. Twenty-one participants were finally recruited, whose demographic data are presented in Table 1. The average age of the participants was 29.57 years, with ages ranging from 18 to 55 years. Regarding gender, 28.5% were men (6) and 71.5% women (15). The average number of years of formal education was 15.33, ranging between 10 and 19 years. All participants voluntarily agreed to be part of the study by signing an informed consent. For statistical analyses, version 26 SPSS software was used. The level of significance was set at 0.05.

**Table 1:** Demographic data of the sample

# Participant	Age	Gender	Level of education (years)
1	25	F	18
2	18	F	13
3	18	F	13
4	25	M	18
5	22	M	13
6	23	M	16
7	19	F	14
8	18	M	13
9	38	F	19
10	19	F	14
11	48	F	17
12	48	F	19
13	23	M	14
14	29	F	17
15	25	F	15
16	55	F	17
17	43	F	19
18	20	M	14
19	52	F	10
20	31	F	13
21	22	F	16
**Mean (SD)**	29.57 (12.37)		15.33 (2.52)

### Procedure

A three-phase process was established to perform the validation of the paradigm. Namely:

- Phase 1. In the initial neuropsychological assessment, the NEUROPSI brief neuropsychological test battery [35] was applied, evaluating verbal and visual memory. To perform that, the word list and semi-complex figure subtests were selected, resulting in the indicators Spontaneous Recall of Verbal Information (SRVI), Cued Recall of Verbal Information (CRVI), Recognition Recall of Verbal Information (RRVI) and Recall of the Semicomplex Figure (RSF), respectively.
- Phase 2. The cognitive training phase was divided into three stages: preparation (relaxation phase), paired-associate training activity and a second training activity based on the MoL.
  - Preparation. It consisted of 2 relaxation sessions through breathing and visualization exercises to improve the participant’s concentration and attention, and it was carried out during the first week for all participants. The reason for using this technique is based on the fact that breathing exercises favor the use of cognitive functions [36].
  - Paired-associate based activity. Prior to the main training activity (MoL-based), we carried out an activity with a lower degree of complexity. In this way, the participant adapts to the cognitive training. This activity is a classic technique that is widely used to diagnose memory deficiencies and also rehabilitate it [37]. The paired-associate training consisted of an activity of coding and retrieval of the corresponding pair element, whose coding is done using the imagination, in the same way that it is done in the MoL philosophy. The level of difficulty depends on the number of pairs to remember and it was applied progressively in 3 levels: 5, 7 and 9 pairs. It should be mentioned that these words do not have any semantic or lexical relationship, because this could change the development of the experiment. Each week the level of difficulty increases each week, and 2 weekly sessions were performed during 3 weeks.
  - MoL-based activity. Training using the MoL consisted of a coding and recovery activity of the elements located in 4 places in the house (Living room, dining room, kitchen and bed room). Coding was done using imagination based on the MoL philosophy. The level of difficulty depends on the number of elements in each place and is was applied progressively in 4 levels, in such a way that 1, 2, 3 and 4 elements were positioned in each of the 4 places. Each week the level of difficulty increased, and 2 weekly sessions were performed during 4 weeks.

The sessions were conducted by the clinical psychologist (and author) Doris Pérez and carried out by a group of 10 psychology students. These sessions were held at participants’ homes to guarantee their attendance, with 2 weekly visits that were scheduled throughout the day (including the night) depending on the availability of the participants.

In short, sessions were held for 2 months, with 2 weekly sessions with each participant. In all the sessions, relaxation and visualization exercises were carried out prior to the execution of the activities. The relaxation sessions lasted exactly 10 min. The paired-associate sessions lasted approximately 10 min, while the MoL sessions lasted approximately 20 min.

- Phase 3. At the end of the intervention, a post test was performed to measure the improvement in verbal and visual memory using the NEUROPSI sub-tests as indicated in phase 1.

### System description and app implementation

**Fig. 1:**
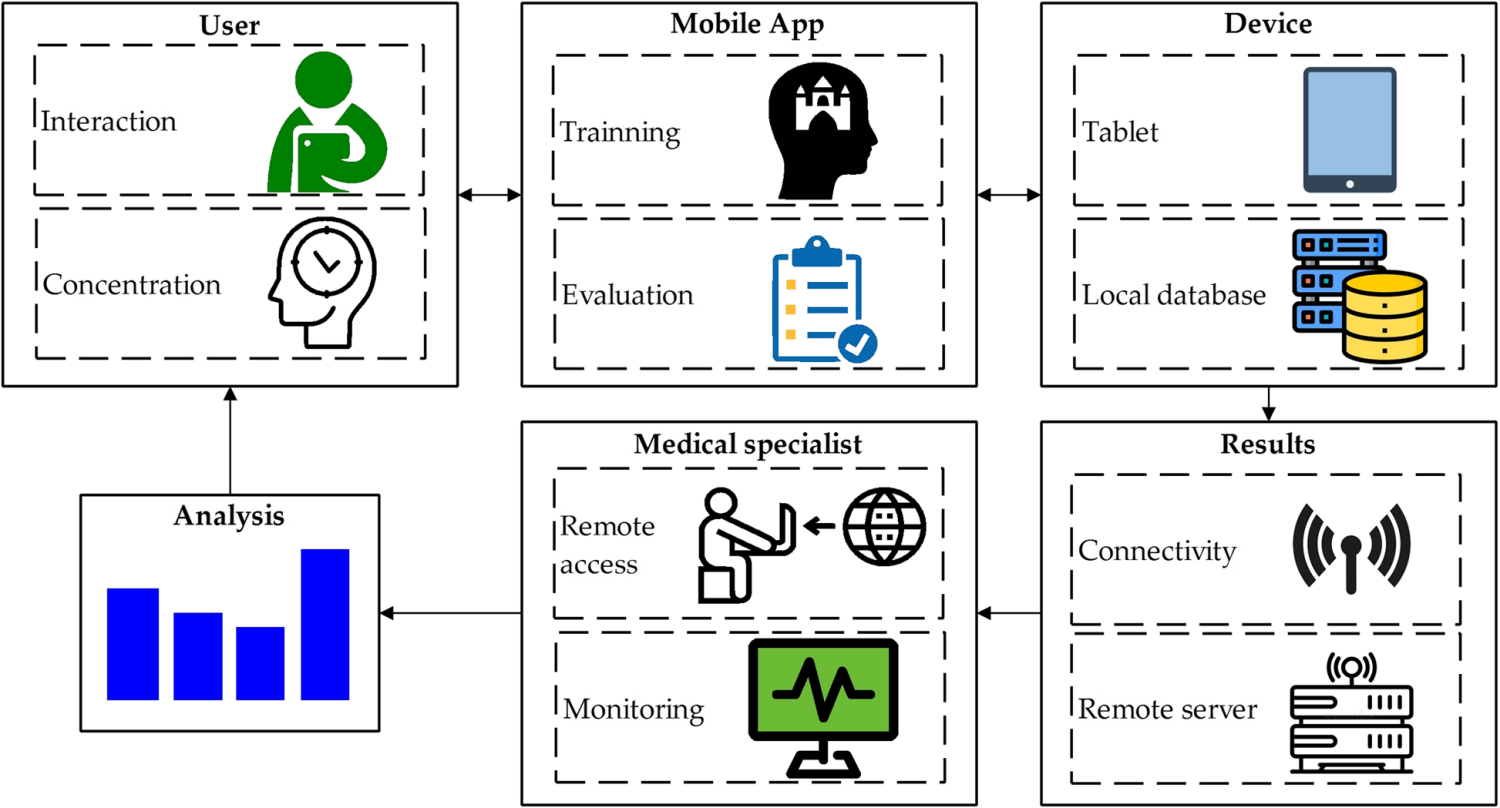
Outline of the implemented methodology

Figure 1 depicts the description of our system. It is based on an application that can be run on any mobile device (either a Tablet PC or a smartphone). Data is stored in a local database and at the same time they are sent to remote server. The medical specialist in charge of the patient can easily monitor the results in a remote way.

The application to collect the data was developed through a user interface developed with Unity software, which was selected to obtain a multiplatform application. The user interface was made up of a single scene that contains the different menus that are activated and deactivated as selected by the user. User interaction with the application is done using buttons and labels, requiring inserting a box collider component to detect the collision with the object. The menus are distributed within a Canvas with text labels and buttons to enter the information, including a keyboard to enter the user’s name. The scripts control the desired actions, manage the interaction between menu items and manage the data collected in the process, including access to the internet to send the results. On the other hand, the collected data is grouped into plain text files and sent to the remote server located at the University of Zaragoza using the SFTP.UploadFile() function of the SSH:NET library. These data can be reviewed from anywhere using sfpt client software using a username and password.

**Fig. 2:**
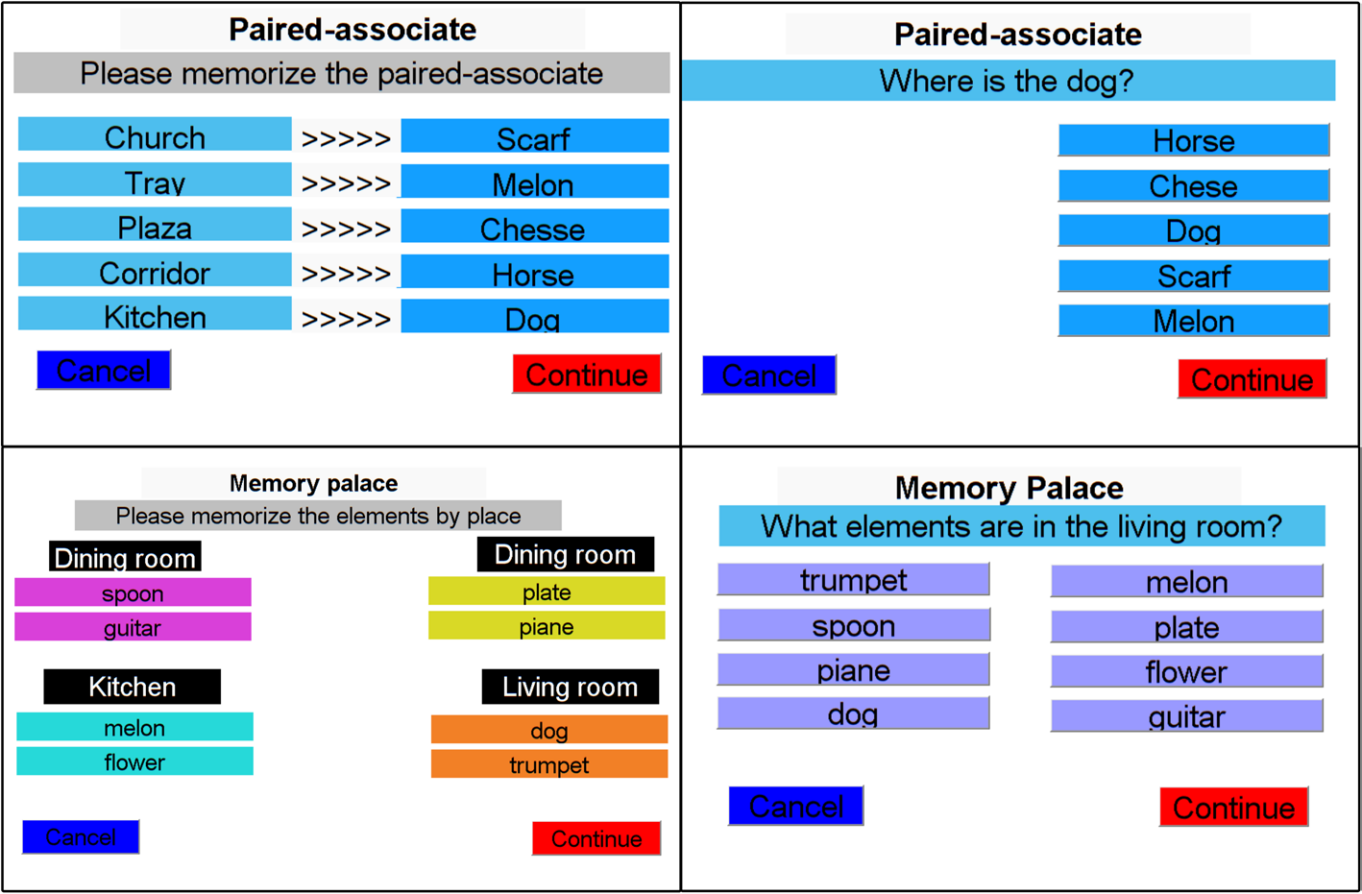
Application screenshots showing the two activities of the cognitive assessment

The activity type and level of difficulty are selected in advance by the user. Figure 2 shows some screenshots of the implemented windows. The paired-associate window shows the list of words to memorize, as well as the options to cancel or continue with the activity. The MoL window shows the 4 places with their respective elements to memorize. In the evaluation phase, the user must select one or more elements, depending on the case.

All activity item names come from a database of 40 randomly selected items. The evaluation window presents the question and the available options are put randomly. Once the evaluation is finished, the initial window is displayed again. The system automatically records the information in spreadsheets, i.e. all the users’ successes and errors to determine AR of every single participant. Similarly, subject’s RT were stored.

**Fig. 3:**
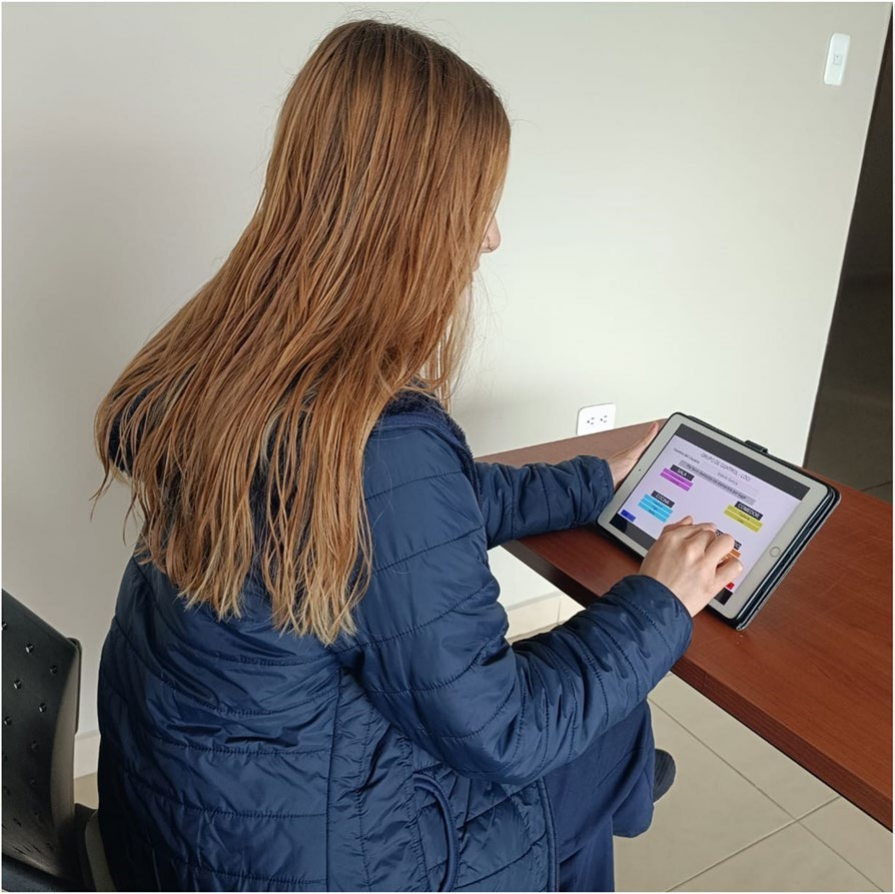
Subject interacting with the app to perform the tasks

The experiments with the participants were carried out on 3 tablets with the following characteristics: Apple iPad, 6th generation, model NUMBER MR7G2TY/A, 9.7-inch LED-backlit Multi-Touch screen with a resolution of 2048 by 1536 pixels, 32 GB storage, A10 Fusion Chip with 64-bit architecture and integrated M10 coprocessor. Equipment with similar specifications was used to ensure that the experiments were carried out under equal conditions. Figure 3 shows a subject performing the tests with the application on a Tablet PC.

## Results

The cognitive assessment and the application of the proposed procedures were carried out at the participants’ homes, respecting the biosafety measures recommended by the World Health Organization (WHO). Two sessions per week were carried out in order to not overload participants. Each session lasted 30 min on average.

### Initial cognitive assessment

Table 2 shows the data obtained after performing the initial cognitive assessment using the NEUROPSI brief battery for each of the participants, together with the mean and standard deviation of the group. These values will serve as a reference to determine the influence of the intervention on the participants.

**Table 2:** Results of the initial cognitive assessment using the NEUROPSI brief battery

# Participant	SVRI	CRVI	RRVI	RSF
1	4	5	6	12
2	5	6	6	12
3	4	2	4	12
4	4	5	6	5
5	5	6	6	6
6	6	5	6	9
7	5	3	6	12
8	6	6	6	11.5
9	5	6	5	12
10	4	5	5	12
11	4	4	4	10
12	4	5	6	11.5
13	5	4	6	12
14	5	5	6	12
15	6	5	6	12
16	3	4	6	4
17	5	4	6	12
18	5	6	5	12
19	5	5	6	9
20	4	6	6	8
21	3	5	6	10
**Mean (SD)**	4.61(0.86)	4.85(1.06)	5.66(0.65)	10.28(2.54)

### Paired-associate activity

**Fig. 4:**
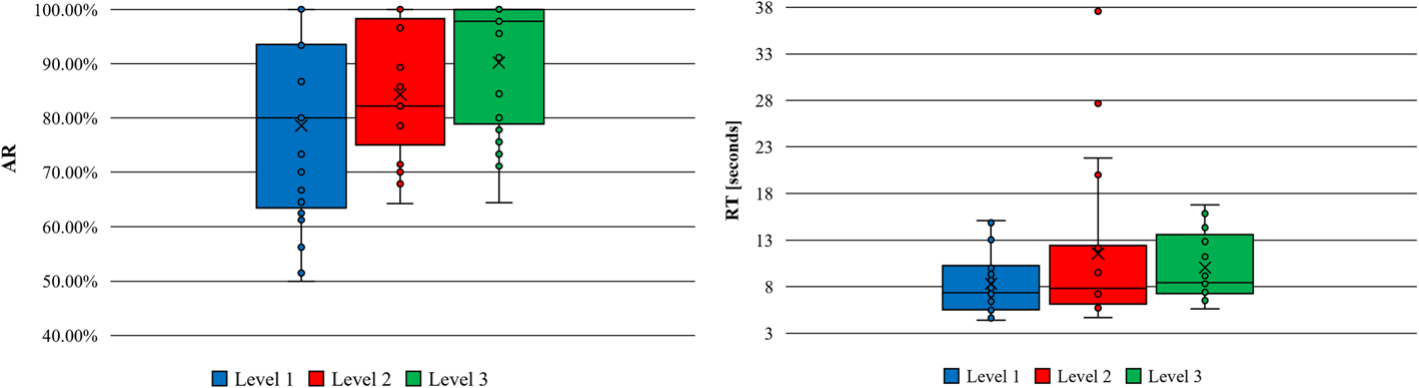
AR and RT results in the paired-associated activity

Table 3 presents the results (mean, median and standard deviation) of the AR and RT for all participants and every single level in the paired-associate activity. Measurements were taken at the end of the different levels. Figure 4 plots the same results.

**Table 3:** Results in the paired-associate activity (AR and RT)

AR				RT		
Level	Mean	Median	SD	Mean	Median	SD
Level 1	78.56%	80.00%	16.45%	8.3096	7.3605	3.2715
Level 2	84.29%	82.14%	11.77%	11.5534	7.8154	8.2103
Level 3	90.16%	97.78%	11.67%	10.0542	8.4556	3.3769

### MoL-based activity

**Fig. 5:**
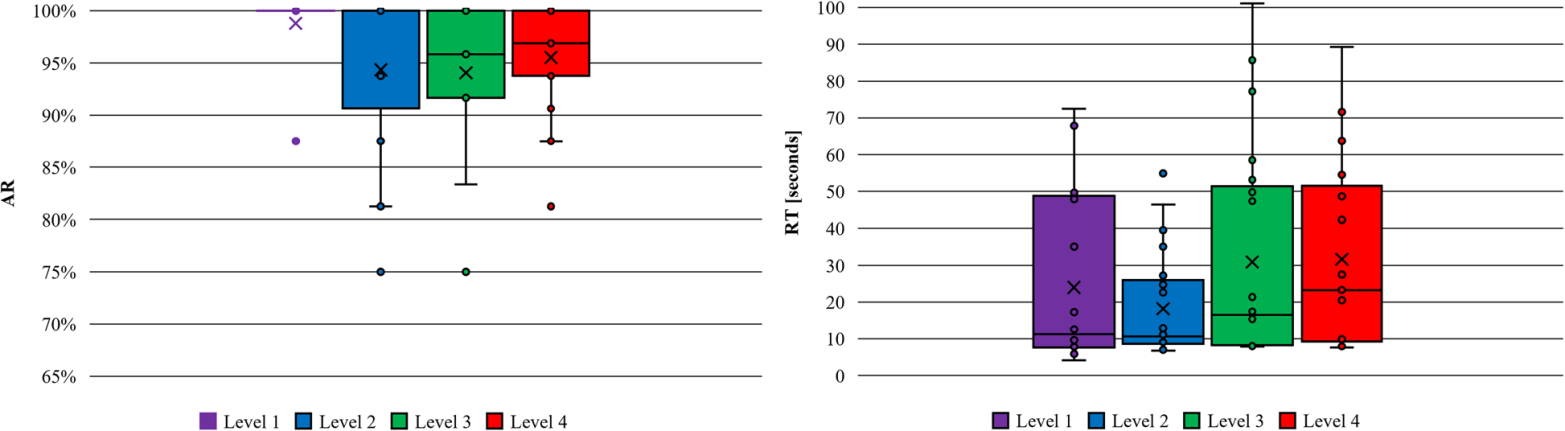
AR and RT results in the MoL activity

Table 4 presents the results (mean, median and standard deviation) of the AR and RT for all participants and every single level in the MoL-based activity. Measurements were taken at the end of the different levels. Figure 5 shows the box-plot diagram with the same results in the MoL activity.

**Table 4:** Results in the activity of MoL (AR and RT)

AR				RT		
Level	Mean	Median	SD	Mean	Median	SD
Level 1	98.81%	100.00%	3.67%	24.0047	11.2810	22.0068
Level 2	94.35%	100.00%	7.45%	18.2025	10.6897	14.0668
Level 3	94.05%	95.83%	7.56%	30.9206	16.5161	28.3947
Level 4	95.54%	96.88%	5.06%	31.6734	23.2769	24.6990

**Fig. 6:**
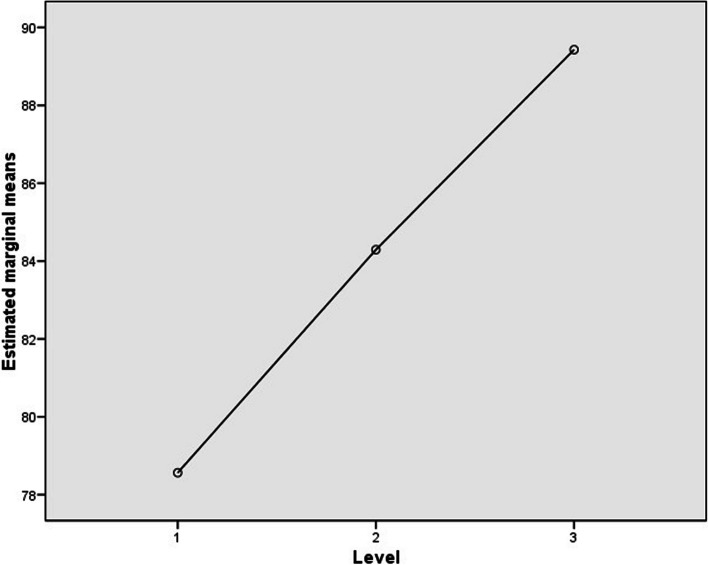
AR estimated marginal means vs. levels in the paired-associate activity

**Fig. 7:**
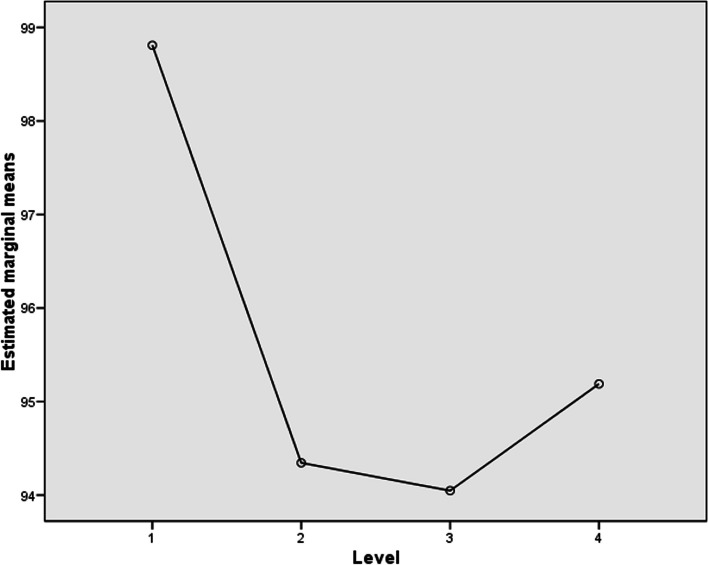
AR estimated marginal means vs. levels in the MoL activity

The intra-subject contrast through an ANOVA of repeated measures revealed that there were significant differences within subjects in the two tasks, the paired-associate learning task (F = 19.11, *P* < .001) and the MoL task (F = 14.359, *P* = .001). The following Figs. (6 and 7) show the marginal means obtained for each of the levels and task, respectively.

### Final cognitive assessment

After the intervention, the participants were again cognitively assessed. Table 5 shows the results obtained in the assessment together with the differences with respect to the initial assessment at baseline.

**Table 5:** Results of the final cognitive assessment using NEUROPSI, together with the difference (d) with respect to the initial assessment (baseline)

# Participant	SVRI	d	CRVI	d	RRVI	d	RSF	d
1	6	+ 2	6	+ 1	6	0	12	0
2	6	+ 1	6	0	6	0	12	0
3	6	+ 2	5	+ 3	6	+ 2	12	0
4	6	+ 2	6	+ 1	6	0	11	+ 6
5	6	+ 1	6	0	6	0	11	+ 5
6	6	0	6	+ 1	6	0	10	+ 1
7	6	+ 1	5	+ 2	6	0	12	0
8	6	0	6	0	6	0	12	+ 0.5
9	6	+ 1	6	0	6	+ 1	12	0
10	6	+ 2	6	+ 1	6	+ 1	12	0
11	6	+ 2	6	+ 2	6	+ 2	12	0
12	6	+ 2	6	+ 1	6	0	12	+ 0.5
13	6	+ 1	6	+ 2	6	0	12	0
14	6	+ 1	6	+ 1	6	0	12	0
15	6	0	6	+ 1	6	0	12	0
16	6	+ 3	6	+ 2	6	0	11	+ 7
17	6	+ 1	6	+ 2	6	0	12	0
18	6	+ 1	6	0	6	+ 1	12	0
19	6	+ 1	6	+ 1	6	0	10	+ 1
20	6	+ 2	6	0	6	0	11	+ 3
21	6	3	6	5	6	0	12	+ 2
Mean (SD)	6 (0)		5.90 (0.3)		6 (0)		11.61 (0.66)	

### Hypothesis verification

To verify our hypothesis, we compared data from the initial (pre-test) and final (post-test) neuropsychological assessments using the Wilcoxon paired signed-rank test. Table 6 shows the contrast results. The results were statistically significant at a significant level α = 0.05, so our hypothesis was validated, and the participants showed a significant improvement in the cognitive assessment carried out after the intervention.

**Table 6:** Paired samples statistics analysis (Wilcoxon signed Rank test)

Pre - posttest differences	Statistic (Z)	Sig.
Spontaneous Recall of Verbal Information (SRVI)	-3.80	0.001
Cued Recall of Verbal Information (CRVI)	-3.51	0.001
Recognition Recall of Verbal Information (RRVI)	-2.07	0.038
Recall of the Semicomplex Figure (RSF)	-2.81	0.005

### Correlation analysis with age and level of education

As a complementary analysis, the degree of association between the results obtained in the two activities (paired associated and MoL) and the age and educational level of the participants was investigated. Pearson’s correlation analysis indicated a moderate-high (*r*=-.486, *p* = .25) and negative relationship between AR and age, although only in the MoL activity. The level of formal education (in terms of years) was not associated with participants’ performance on both tasks, so performance did not depend on educational level. The degree of association between the performance of the two tasks is very high (*r* = .771) according to Cohen [38]. Regarding RT, we did not find any association with years of education. However, we found a positive association between age and RT in the MoL activity (*r* = .477, *P* = .029). Regarding the subscales (SRVI, CRVI, RRVI, RSF) of the NEUROPSI test, neither age nor level of education were associated (Spearman’s r not significant) with the results in these subscales neither in the pretest nor in the post test.

The t-student analysis revealed no significant differences by sex in the AR neither for the paired associate task (t_19_ = 0.909, *P* = .375) nor for the MoL task (t_19_ = 0.352, *P* = .729), respectively. There were also no significant differences by gender in RT neither in the paired associate task (t_19_=-0.470, *P* = .643), nor for the MoL task (t_19_=-0.283, *P* = .780), respectively. Finally, none of the subscales (SRVI, CRVI, RRVI, RSF) revealed differences by gender in the post test. In the pretest, only significant differences were found by sex in the MVE (t_19_ = 3.83, *P* = .001).

## Discussion

This research was designed based on a mnemonic tool present since classical times. Unlike the pencil and paper-based techniques, we offer an app-based variant that can run on both a smartphone and a Tablet PC and it is therefore a low-cost solution. The results obtained in the evaluations (pre and posttest) have confirmed our hypothesis, and the participants obtained a better (and significant) cognitive assessment in all the subscales of the NEUROPSI brief neuropsychological test battery [35] after 2 months of intervention. With our computerized proposal, data were collected automatically and stored in separate files for subsequent analysis, avoiding manual data transcription. With this work we have also shown that a very simple implementation, away from complex VR environments, can be used for cognitive training and rehabilitation. The portability of the developed tool makes it ideal for daily cognitive practice at home-based environments.

We have found in literature several proposals that use the pairing of elements as methods to exercise memory [39, 40, 41]. The novelty of our study is that this method was used as a preparatory and gradual exercise that precedes another cognitive training tool (MoL). The difficulty of the MoL is more than evident, and some authors have pointed out the great cognitive effort that this technique entails [30], so it seems appropriate to make a prior approach to this demanding method. Our research distinguishes itself from previous studies [7, 25, 29] by introducing a practical and accessible solution for cognitive training through a mobile application. Thus, the traditional use of pencil and paper is left behind, proposing a methodology that integrates a preparation task followed by the implementation of the computerized MoL. This approach has shown significant improvements after two months of intervention.

Compared with research that has focused on virtual reality (VR) techniques [30–32], our contribution focuses on the effectiveness of the MoL as a mnemonic strategy in an accessible non-immersive digital environment. Unlike the study [30], our work introduces a preparatory stage before the MoL and, unlike [31] and [32], our study extends the application of the MoL beyond the educational context and offers a more comprehensive assessment of its effectiveness. Our study broadens the field of cognitive training by providing a digitized and accessible solution, which demonstrates the efficacy of the MoL and highlights the importance of a preparatory stage. Due to the demanding nature of the MoL method, we consider that it is pertinent to gradually introduce it to the participants. The objective is to establish the necessary bases so that they can tackle activities that represent a greater challenge for memory. Also, no other cognitive exercise was included because the evidence presented by Li et al. [42] suggests that training focused exclusively on memory may produce better results than a combined approach, which would involve both executive function training and memory strategy training.

### Spatial and navigational metaphor and MoL

Episodic memory theories suggest that spatial context plays a fundamental role in episodic memory. Robin et al. [43] in their study and with the help of Functional Magnetic Resonance Imaging (FMRI), suggested that the spatial context of an event plays an important role in how it is represented in the brain. Also Reggente et al. [44] in their study, showed evidence that spatial contexts are important in the coding process. The study suggested that if one tries to memorize an object in one spatial location, the chances of remembering it will be higher. Since the MoL precisely enforces that object-location binding in the encoding process, if this feature is removed its effectiveness may be compromised.

In our case though, although we have not made a comparison with a control group using a technique that reinforces this spatial component, the results have been good after the intervention period. This is an idea that is still on the table, since there are MoL implementation studies [7] like ours, in which this spatial component in coding does not seem to be of vital importance. The study of Sandberg et al. [7] also implemented a MoL in a smartphone application without using virtual environments or navigation strategies typical of these environments. After an intervention of 3 months, the results revealed that the level reached in the face-name paired associates cue recall memory test (transfer test to check proficiency in MoL) depended on the dose, in such a way that those practicing more had better proficiency in MoL. Another interesting result coming from that study was that half of the participants benefited in everyday life from learning and practicing with the MoL. In fact, Caplan et al. [25] conclude that, despite the popularity of the MoL, its effectiveness may not critically depend on imagined navigation, being able to better resemble traditional peg-based strategies that do not include navigation. Results like that of Legge et al. [45] support this last theory, since they observed in their study an equivalent memory performance for the participants regardless of the medium in which the MoL was administered (virtual versus conventional).

The implementation of the MoL in VR environments has put on the table the influence of the levels of immersion with which we provide VR. Nevertheless, there is still a lot to work to do on this idea. For example, Ragan et al. [46] in their study managed to obtain empirical evidence that higher levels of immersion can produce an improvement in performance when it comes to an abstract mental activity. They also pointed out, in line with the above, that the performance improvements caused by a higher level of immersion can be attributed to enhanced spatial cues. However, an added problem when MoL is implemented in virtual reality environments arise. Some studies have dealt with the difficulty in navigation by the participants [29, 32]. Vindenes et al. [32] in their implementation by means of immersive VR reported precisely this difficulty in navigation by the participants, as well as low spatial ability. This fact caused a worse performance in those participants who used the immersive version of VR compared to those who used a desktop application. It must also be taken into account that not all people have a good predisposition for the use of VR environments and therefore whether their use will generate better results or not must be analyzed. In our case, the activity was implemented in a non-immersive environment and was very guided, so there were no problems with navigation. It is also noteworthy that the aforementioned proposals did not introduced introductory techniques to the MoL as we did.

### Age-related effects

Regarding the well-known age-related effects observed on episodic memory tasks, in our case we have been able to verify these negative effects on performance as age increases. These results are in line with other studies that have implemented the MoL-based tasks [47], as well as tasks for the evaluation of episodic memory in virtual environments [48, 49, 41]. Also, in Gross et al.’s study [29], all groups showed some form of age-related decline in memory performance. The mean age of the participants was very low in our case, which may be a plausible explanation as to why this phenomenon was only observed in the MOL task, but not in the paired-associate task. Regarding RT, we found a positive association between age and reaction time in the MoL activity. This observed correlation is in line with studies that have been using tasks to measure episodic memory, such as that conducted by Varela et al. [50], Armstrong et al. [51] or Ouellet et al. [48].

### Limitations

Our study presented some limitations that deserve to be considered for a more complete understanding of the results and their context. First, the selection of the participants was made from a group of volunteers. This decision could have generated a selection bias, since it is possible that people with a good memory and a particular interest in technological applications were more interested in participating. No control groups, neither passive nor active, were included in the design of the experiment, so all the participants were part of the experimental group, limiting the interpretation of our results. Another limitation to take into account is the size of the sample, made up of 21 participants, which could limit the generalization of the findings to a broader population. Likewise, follow-up tests were not carried out nor was the transfer of the knowledge acquired to the real life of the participants evaluated, two important aspects that can affect the practical application.

In addition, the implemented interventions were not personalized for the participants, which may have influenced the effectiveness. Developing applications that are adapted to the user is an aspect that requires a high level of technological development. Finally, although the intervention period was adequate in general terms, it is plausible that an increase in the dose of the intervention could have led to better performance in the proposed tasks. We acknowledge the existence of these limitations and consider them valuable indications for future research. Based on this learning, one of our main goals is to develop a Randomized Controlled Trial (RCT) in future studies to mitigate these limitations and improve the robustness of the findings.

### Applications and future work

As future work, the authors plan to continue developing technological applications for the diagnosis, training and rehabilitation of human cognition. As a first objective, we intend to take this methodology to the field of cognitive telerehabilitation by automating the process of sending data periodically to the therapists, so they can evaluate the cognitive status of each patient and their evolution. Likewise, the creation of tools for the remote guidance of the intervention by neuropsychologists. With all this we can not only respond to the physical distancing effects but we can also pave the way to a mixed model in health care where hospital care is complemented with telerehabilitation procedures [52]. In this way, a cost-effective and sustainable paradigm for the post-pandemic era can be created [53].

The next step is the application of this research to patients with a diagnosed cognitive impairment, in order to evaluate the results obtained. We seek to apply our application with participants with cognitive defects such as parkinsonism, autism, Alzheimer’s, and so forth. For example, some studies have already shown that suitable computerized game-based solutions can improve cognition in children with autism [54, 55]. The design of goal-oriented memory strategy programs can significantly improve cognitive functioning as well as performance of activities of daily living and quality of life in people with cognitive impairments [56]. The generalization of results must be carried out through RTC designs, once the pandemic situation ends.

An important line of research that we want to pursue is whether the improvements presented by the participants can have a positive transfer to the performance of Activities of Daily Living (ADLs) for this type of patients. Finally, and given that there is not enough evidence to date, we intend to migrate our application to both immersive and non-immersive virtual reality environments, to elucidate if they really provide tangible benefits in the field of cognitive rehabilitation. Many authors claim an important role for this technology applied through active video games as an ideal therapeutic modality in a global pandemic environment [57]. There are other studies, such as the conducted by Kim et al. [58] in which the combination of VR training and computer-based cognitive rehabilitation could add benefit is for the treatment of cognitive impairment in stroke patients. In this sense, the level of immersion in VR can play an important role in learning. Huttner and Robra-Bissantz [59] found improvements of 5–6% for the immersive VR group versus the computer screen group in an immersive virtual reality MoL implementation. Some results found in the study of Ragan et al. suggested that the increase of the level of immersion (such us the level found in Head-Mounted Displays (HMD)) can improve performance in a significant way in cognitive tasks compared to lower levels of immersion. However, higher levels of immersion do not necessarily lead to higher cognitive performance, so many more studies are needed to find evidence of the role played by the level of immersion [60]. On the other hand, the study of Blunt and VanArsdall [61] pointed out towards an improvement in retention for the MoL when animacy and animate imagery are used. The aforementioned ideas open up interesting lines of work based on the joint interaction of several computerized cognitive strategies as well as on the enrichment of virtual systems to favor cognitive performance.

## Conclusions

With this work, we have validated a cognitive training methodology based on a preparatory task prior to a mnemonic tool (MoL) that requires a great cognitive effort. The combination of both methods and the way of administering them has achieved positive and statistically significant results as far as cognitive performance in memory tasks in a sample of adults. One of the main advantages of our work lies in its simplicity, since costly and complicated implementations are not necessary to achieve benefits in the field of cognitive rehabilitation. In situations of forced social isolation such as the current COVID-19 pandemic, the need to implement strategies that allow the retraining of cognitive functions is evident. These strategies must be based on studies that show that their application generates real improvement in the patient. The so developed application can contribute to the rise of telehealth and telemedicine through wearable devices for screening and surveillance of patients suffering from any cognitive impairments.

## Data Availability

The datasets generated during the current study are available from the corresponding author on reasonable request.

## Abbreviations

ADL: Activity of Daily Living
AR: Accuracy Response
CRVI: Cued Recall of Verbal Information
CVA: Cerebrovascular accident
HMD: Head-mounted display
MCI: Mild Cognitive Impairment
MoL: Method of Loci
MRI: Magnetic Resonance Imaging
NEUROPSI: Neuropsychological test battery
RRVI: Recognition Recall of Verbal Information
RSF: Recall of the Semicomplex Figure
RT: Reaction Time
SRVI: Spontaneous Recall of Verbal Information
VR: virtual reality
WHO: World Health Organization
RCT: Randomized Controlled Trial
